# Risk factors for revision due to infection after primary total hip arthroplasty: A population-based study of 80,756 primary procedures in the Danish Hip Arthroplasty Registry

**DOI:** 10.3109/17453674.2010.519908

**Published:** 2010-10-08

**Authors:** Alma B Pedersen, Jens E Svendsson, Søren P Johnsen, Anders Riis, Søren Overgaard

**Affiliations:** ^1^Department of Clinical Epidemiology, Aarhus University Hospital, Århus; ^2^Department of Orthopaedic Surgery and Traumatology, Clinical Institute, Odense University Hospital, University of Southern Denmark, Odense, Denmark

## Abstract

**Background and purpose:**

There has been a limited amount of research on risk factors for revision due to infection following total hip arthroplasty (THA), probably due to low absolute numbers of revisions. We therefore studied patient- and surgery-related risk factors for revision due to infection after primary THA in a population-based setting.

**Materials and methods:**

Using the Danish Hip Arthroplasty Registry, we identified 80,756 primary THAs performed in Denmark between Jan 1, 1995 and Dec 31, 2008. We used Cox regression analysis to compute crude and adjusted relative risk (RR) of revision due to infection. Revision was defined as extraction or exchange of any component due to infection. The median follow-up time was 5 (0–14) years.

**Results:**

597 primary THAs (0.7%) were revised due to infection. Males, patients with any co-morbidity, patients operated due to non-traumatic avascular femoral head necrosis, and patients with long duration of surgery had an increased RR of revision due to infection within the total follow-up time. A tendency of increased RR of revision was found for patients who had received cemented THA without antibiotic and hybrid THA relative to patients with cementless implants. Hip diagnosis and fixation technique were not associated with risk of revision due to infection within 1 year of surgery (short-term risk).

**Interpretation:**

We identified several categories of THA patients who had a higher risk of revision due to infection. Further research is required to explain the mechanism underlying this increased risk. More attention should be paid by clinicians to infection prevention strategies in patients with THA, particularly those with increased risk.

As with any other surgical operation, serious complications in patients undergoing total hip arthroplasty (THA) include infections. Most infections stem either from contamination in the operating room or from later hematogenous spread. Deep infection is the third most common cause of revision of THAs in Denmark (DHR Annual repport 2008). In the last 2 decades, advances in theater design and the prophylactic use of antibiotics, either systemically or incorporated in cement, have substantially reduced the incidence of infection after hip replacement (Zimmerli and Ochsner 2003, Ridgeway et al. 2005, Phillips et al. 2006). However, recent studies in the United States and Norway have found increasing infection rates (Dale et al. 2009, Kurtz et al. 2010).

Research on risk factors for revision due to infection following THA has been limited, probably due to low absolute numbers of revisions. However, in the last few years several reports have suggested that some patient- and surgery-related factors may play a role (Furnes et al. 2001, Saleh et al. 2002, Ridgeway et al. 2005, Engesaeter et al. 2006, Bongartz et al. 2008, Pulido et al. 2008, Dale et al. 2009, Hooper et al. 2009, Ong et al. 2009). Comparison of these studies is difficult due to different inclusion criteria for the study population and different definitions of infection, sometimes including both joint infections and superficial infections, or infections in general. We studied only the infections that were followed by revision of the implant.

For this reason, we conducted a nationwide follow-up study using the Danish Hip Arthroplasty Registry to examine potential patient- and surgery-related risk factors for revision due to infection.

## Material and methods

### Data sources and settings

We designed the study as a population-based follow-up study using data from the prospective, nationwide Danish medical registries. Since 1968, all Danish citizens have been assigned a unique 10-digit personal identification number, thus permitting unambiguous linkage of records in different databases (Pedersen et al. 2006). The Danish National Registry of Patients (NRP) holds information about all contacts with public hospitals in Denmark since 1977, including dates of all admissions and discharges and with up to 20 diagnoses for each admission. The diagnoses are classified according to the International Classification of Diseases (eighth edition (ICD-8) up to 1993 and the tenth edition (ICD-10) after that).

### Study population

The Danish Hip Arthroplasty Registry (DHR) was established on Jan 1, 1995 and is a nationwide clinical database of all primary THAs and revisions performed in Denmark. Registration in the DHR is compulsory. All orthopedics departments performing total hip replacement, including private hospitals (n = 52), report pre- and peroperative data from both primary and revision surgery to the registry using a standard registration form. We identified all primary THA procedures in the DHR (n = 80,756) performed from Jan 1, 1995 to Dec 31, 2008. Of these, 12,234 (15%) were bilateral procedures (including 758 one-stage and 11,476 two-stage procedures).

### Risk factors

As possible patient-related risk factors, we considered age (10–59 years, 60–69 years, 70–79 years, and 80+ years), sex, indication for primary THA (primary osteoarthritis (OA), sequelae from trauma (including both THA due to acute proximal femoral fracture and sequelae from the same one), non-traumatic avascular femoral head necrosis (AVN), inflammatory arthritis, and other hip diseases), and previous surgery on the same hip. Based on the NRP, for each patient we assessed the co-morbidity level using the Charlson co-morbidity index (Charlson et al. 1987), which was originally developed and validated for the prediction of short- and long-term mortality in patients admitted to a department of internal medicine (Deyo et al. 1992) and adapted for use with hospital discharge registry databases (De Groot et al. 2003). The Charlson index includes 19 major disease categories extracted from the NRP using all primary and secondary discharge ICD-8 and ICD-10 diagnoses for all hospitalizations and outpatient visits from January 1, 1977 to the date of primary THA. A weight of 1, 2, 3, or 6 points is assigned to discharge from each co-morbidity category and the index score is the sum of these weights. We classified the patients according to 2 levels of co-morbidity: low (score 0 at the time of surgery) and medium/high (score ≥ 1).

Data regarding fixation technique (cemented with antibiotics, cemented without antibiotics, cementless, or hybrid), duration of surgery (less than 60 min, 61–90 min, 91–120 min, or more than 120 min), type of anesthesia (general or regional), operating theater (conventional or laminar-flow ventilation) and ossification prophylaxis with NSAID were considered as prosthetic and surgery- related risk factors. Furthermore, calendar year of surgery was investigated as a risk factor. Information on all these risk factors was obtained from the DHR. Different types of previous surgery and systemic antibiotics were not studied as risk factors due to the insufficient sample size and low number of revisions.

### Outcome

The outcome was time to failure, i.e. first revision due to infection. Revision was defined as a new surgical procedure involving partial or complete removal or exchange of any of the components. Infection was diagnosed by the surgeon reporting to the registry.

### Statistics

The follow-up period started on the day of primary THA and ended on the day of revision, death, emigration, or Jan 1, 2009, whichever came first. We used Cox's regression analysis to examine the association between possible risk factors and risk of revision due to infection We estimated hazard ratio as a measure of relative risk (RR) with 95% confidence interval (CI) for each risk factor, crude and mutually adjusted for all risk factors. We studied the revision risk during the entire follow-up time period, and also the revision risk within 1 year of THA surgery (short-term risk). Bilateral THAs were treated as 2 independent observations according to previous findings of no difference in RR estimates irrespective of whether the bilateral THA was included or excluded from the analysis (Lie et al. 2004).

All analyses were performed using a statistical software package (SAS version 9.1.3; SAS Institute Inc, Cary, NC). Assumption of proportionality of the Cox regression model was assessed graphically for each risk factor and found appropriate.

The study was approved by the Danish National Board of Health and the Danish Data Protection Agency (J. no. 2007-41-0720).

## Results

We identified 80,756 primary THAs for the study in the DHR during the study period, which had been performed on 74,639 patients. Thus 6,117 patients (corresponding to 12,234 THAs) received THAs in both hips. 46,831 THAs (58%) were in females, 28,729 THAs (36%) had been associated with prior co-morbidities, and 63,318 THAs (78%) were operated due to primary OA. During the maximum follow-up time of 14 years (median 4.6 years), 597 primary THAs (0.7%) were revised due to infection: 149 (25%) involved total exchange of the prosthesis, 146 (24%) involved partial exchange of the prosthesis, and 302 (50%) involved total removal of the prosthesis.

### Patient-related risk factors for revision due to infection (Table 1)

Male sex was associated with an increased adjusted RR of revision, whereas age had no influence on the risk. Patients with AVN as primary hip diagnosis had an increased adjusted RR of revision compared to patients with primary OA, whereas patients with inflammatory arthritis and other hip diagnoses had the same risk as OA patients. There tended to be more infections in patients with proximal femoral fracture. The presence of any co-morbidity at the time of surgery was found to be a predictor of revision. Previous surgery on the same hip did not affect the revision risk. Among 9,321 patients with previous surgery on the same hip, three-quarters were registered as osteosynthesis and the rest as hemiarthroplasty, surgery due to acetabulum fracture, proximal femoral osteotomi, or other surgery. The material was too small to study the risk of revision due to infection separately for different types of previous operations.

**Table 1.** Patient-related risk factors for revision due to infection following primary total hip arthroplasty during the maximum follow-up time of 14 years

	Patients (n)	Revision due to infection (n/%)	Crude RR (95% CI)	Adjusted RR **^a^** (95% CI)
Sex				
Female	46,831	293 (0.63%)	1 (ref.)	1 (ref.)
Male	33,925	304 (0.90%)	1.46 (1.24–1.71)	1.53 (1.30–1.80)
Age groups (years)				
0–59	16,537	126 (0.76%)	1 (ref.)	1 (ref.)
60–69	24,626	183 (0.74%)	1.01 (0.80–1.26)	0.98 (0.77–1.24)
70–79	27,422	210 (0.77%)	1.06 (0.85–1.32)	0.97 (0.75–1.26)
80+	12,171	78 (0.64%)	0.97 (0.73–1.29)	0.86 (0.63–1.19)
Charlson co-mobidity index				
Index, low (0)	52,027	356 (0.68%)	1 (ref.)	1 (ref.)
Index, medium to high (1+)	28,729	241 (0.84%)	1.38 (1.17–1.63)	1.30 (1.09–1.54)
Diagnosis for primary THA				
Primary osteoarthrosis	63,318	443 (0.70%)	1 (ref.)	1 (ref.)
Proximal femoral fracture	9,380	88 (0.94%)	1.51 (1.20–1.90)	1.46 (0.99–2.17)
Non-traumatic AVN	2,148	28 (1.30%)	1.85 (1.26–2.71)	1.70 (1.15–2.53)
Inflammatory arthritis	2,067	21 (1.02%)	1.33 (0.86–2.06)	1.19 (0.76–1.88)
Other diagnoses	3,843	17 (0.44%)	0.63 (0.39–1.03)	0.63 (0.38–1.04)
Previous surgery to the same hip				
No	71,435	513 (0.72%)	1 (ref.)	1 (ref.)
Yes	9,321	84 (0.90%)	1.32 (1.05–1.67)	0.95 (0.64–1.41)

**^a^** Relative risk mutually adjusted for other factors in Tables 1 and 2, and calendar year of surgery.

**Table 2.** Prosthesis- and surgery-related factors and calendar year of surgery as risk factors for revision due to infection following primary total hip arthroplasty during the maximum follow-up time of 14 years

	Patients (n)	Revision due to infection (n/%)	Crude RR (95% CI)	Adjusted RR ^**a**^ (95% CI)
Fixation technique				
Cementless	25,571	132 (0.52%)	1 (ref.)	1 (ref.)
Cement with antibiotics	25,461	184 (0.72%)	1.25 (1.00–1.56)	1.24 (0.94–1.62)
Cement without antibiotics	9,185	96 (1.05%)	1.38 (1.06–1.81)	1.41 (1.01–1.96)
Hybrid	20,539	185 (0.90%)	1.47 (1.18–1.84)	1.53 (1.19–1.96)
Duration of surgery (min)				
≤ 60	24,511	130 (0.53%)	1 (ref.)	1 (ref.)
61–90	37,273	283 (0.76%)	1.23 (1.00–1.52)	1.14 (0.91–1.42)
91–120	13,608	103 (0.76%)	1.22 (0.94–1.58)	1.09 (0.83–1.44)
121+	5,283	81 (1.51%)	2.29 (1.73–3.02)	2.02 (1.49–2.75)
Ossification prophylactic treatment				
No prophylaxis	70,993	512 (0.72%)	1 (ref.)	1 (ref.)
NSAID treatment	9,763	85 (0.87%)	1.04 (0.83–1.31)	1.05 (0.83–1.34)
Type of anesthesia				
Regional	61,287	427 (0.70%)	1 (ref.)	1 (ref.)
Universal	19,469	170 (0.87%)	1.22 (1.02–1.46)	1.17 (0.97–1.40)
Operating theater				
Conventional ventilation	8,333	80 (0.96%)	1 (ref.)	1 (ref.)
Laminar air flow ventilation	72,423	517 (0.71%)	0.81 (0.64–1.03)	0.90 (0.70–1.14)

**^a^** Relative risk mutually adjusted for other factors in Tables 1 and 2, and calendar year of surgery.

### Prosthetic concept and surgery-related risk factors for revision due to infection (Table 2)

Patients with hybrid THA and cemented THA without antibiotics had an increased adjusted RR of revision compared to patients operated with cementless THA. There was an association between cemented THA with antibiotics and elevated risk of revision, but this did not reach statistical significance. Surgery exceeding 2 h was associated with an elevated risk of revision due to infection relative to patients operated for less than 1 h. We found no association between ossification prophylaxis, type of anesthesia, or type of operating theater on the one hand and risk of revision on the other.

Compared to the period 1995–1997, the risk of revision was similar for patients operated in 1998–2002 (adjusted RR = 0.94, CI: 0.74–1.18) and in 2003–2005 (adjusted RR = 0.85, CI: 0.63–1.13). In contrast, the risk of revision was 1.45 times higher (CI: 1.07–1.96) for patients operated in 2006–2008 compared to those operated in 1995–1997.

### Risk of revision due to infection within 1 year of THA surgery: subanalyses

During the first year after THA surgery, male sex, any co-morbidity, and the period 2006–2008 (as compared to 1995–1997) were risk factors for revision similar to those found during the entire follow-up period. AVN hip diagnosis and fixation technique, which were risk factors during the entire follow-up period, were not risk factors during the first year (data not shown).

## Discussion

In this observational, nationwide population-based study of 80,756 THA procedures, 0.7% were revised due to infection. We identified several patient-, prosthesis-, and surgery-related risk factors for revision that might be considered with a view to improving infection strategies.

The strengths of our study include its population-based, prospective design with a large sample size and complete follow-up. Furthermore, the validity of our findings also depends on accurate coding of data in the DHR, as well as completeness of registration of both primary THA and revision cases in the DHR. We have previously documented that overall, there is a high validity of data in the DHR and a completeness of registration of approximately 94% (Pedersen et al. 2004). Due to the prospective registration of data in DHR, it is highly unlikely that any missing registrations of the primary THA are systemtically linked to later revision status. Thus, lack of registration of revisions may cause underestimation of the overall revision rate in our study, but it would not have biased relative risk estimates substantially. In addition, we believe that nowadays most of the cases with acute infection will either have revision surgery or a debridement with exchange of some components (liner and/or head).

Several limitations of the study must also be pointed out. Lack of registration with the NRP and inconsistent coding practices may have influenced estimation of the Charlson co-morbidity index, and thereby introduced residual confounding in multivariate analyses. Misclassification of the Charlson index may lead to information bias if it is related to revision. However, the latest validation study of the diagnoses included in the Charlson co-morbidity index, as ascertained from the NRP, showed a high quality of hospital discharge data for all 19 diagnoses (Thygesen et al. 2009).

We were able to adjust for a number of possible confounders. However, some other confounders related to risk factors and infection rate may have influenced our risk estimates, e.g. patient weight, height, smoking, medication at follow-up, and alcohol intake. This information was not available in our dataset. Finally, we should bear in mind that the absolute risk of revision is low when interpreting relative risks.

### Patient-related risk factors

A few other studies (Malchau et al. 2002, Dale et al. 2009, Jämsen et al. 2009, Ong et al. 2009, Kurtz et al. 2010) have also found that males have a higher risk of long-term total hip and knee arthroplasty failure due to infection. THA in males often means a greater degree of surgical trauma and tissue necrosis than in females. It is possible that male patients have a greater chance of being referred to an orthopedic specialist due to infection (Franks and Clancy 1997) or that surgeons have lower thresholds for males than for females when deciding on indications for revision (Hawker et al. 2000). Our finding of increased infection risk in THAs with a Charlson score of ≥ 1 is not surprising, since a number of diseases included in the Charlson co-morbidity index such as stroke, liver disease, diabetes, and cancer are known to be associated with poor bone quality and increased mortality rate (Arikoski et al. 1999, Levendoglu et al. 2004, Schwartz et al. 2005). Kurtz et al. (2009) found that the presence of pre-existing co-morbidity in terms of the Charlson index score being ≥ 1 increase the risk of prosthetic hip infection (p < 0.0001) and the adjusted RR for a Charlson score of 5+ as compared to a score of 0 was 2.2.

Regarding primary diagnosis, AVN was a strong predictor of long-term risk of THA infection, but not for short-term risk. Similar failure rates in general between AVN patients and standard THA patients have been reported (Myers et al. 2010). However, revision due to infection has been found to be commoner in AVN patients than in OA patients (Havelin et al. 2000, Malchau et al. 2002, Radl et al. 2005). The increased long-term risk of infection in AVN patients may be due to underlying risk factors present in the AVN population but not in the OA population, such as corticosteroid therapy, trauma, renal disease, and alcohol consumption, which together cause 90% of cases (Mont and Hungerford 1995). These factors are associated with increased risk of infection, and they may thereby introduce the confounding bias in our estimates. Unfortunately, there was no information on these factors available in our dataset, or in previously reported studies. The similar short-term risks of revision due to infection in AVN and OA patients may suggest that pre-, peri-, and immediate postoperative care of AVN patients was similar to, if not better than, that of OA patients.

Previous studies have reported an increased risk of joint infection in hip and knee replacement patients with inflammatory arthritis (Bongartz et al. 2008, Jämsen et al. 2009), which was not confirmed in our study. A study by Bongartz et al. (2008) found a 4 times higher risk of joint infection in rheumatoid arthritis patients than in OA patients in a small population of 462 hip and knee patients, which introduced uncertainty in their estimate with a wide confidence interval. In addition, they studied not only revisions due to infection but also infections treated nonoperatively conservative, making comparison with our estimates difficult. Jämsen et al. (2009) used the same outcome as we did and included a large patient sample, but the study was based on knee replacements, which might have had a different risk of revision than hip replacement patients. Even so, our results agree with the results from the latest large registry-based study on hip replacement patients (Dale et al. 2009).

### Prosthesis- and surgery-related risk factors

The increased long-term risk of revision due to infection in hybrid and cemented implants than in uncemented ones is in accordance with the results from the New Zeeland hip register (Hooper et al. 2009). In our study, the risk of infection in cemented implants was influenced by high risk in cemented implants without antibiotics, as has been reported previously (Engesaeter et al. 2006, Hooper et al. 2009, Jämsen et al. 2009). Thus, in cemented primary THAs, cement with antibiotic should be used. Nevertheless, the opposite tendency was seen in Norway (Dale et al. 2009), where an increased risk of revision due to infection in uncemented implants was seen compared to cemented implants with antibiotics. Since literally all of our THA patients received systemic antibiotic, this could not have been the reason for the discrepancy. It has been tradition to use uncemented implants in Denmark for many years, to a much higher extent than in Norway. Long-term, the risk of revision for any reason in uncemented implants has been reported to be 1.6 and 2.1 times higher in Norway and Sweden, respectively, than in Denmark (Havelin et al. 2009). However, the risk of revision due to infection has not yet been studied in the Nordic Arthroplasty Register Association (NARA). We have no clear explanation for the increased risk of long-term revision in hybrid implants, since more than 85% of hybrids included cement with antibiotic and all received systemic antibiotic. One can speculate that the cement itself may be a source of microorganisms (Tunney et al. 2007).

One interesting finding was that the risk of infection within 1 year was similar for different fixation techniques, suggesting that the long-term risk of infection may have something to do with patient characteristics, the implant brand used, or cement-related factors, as mentioned above; this requires further study.

We found an association between long duration of surgery and risk of revision, which agrees with previous findings (Kessler et al. 2003, Smäbrekke et al. 2004, Ong et al. 2009). Some researchers have suggested that each additional minute of operating time leads to a 3% increase in perioperative complications (Kessler et al. 2003). Long procedures can also increase the risk of blood loss, hematoma formation, or venous thromboembolism (Jibodh et al. 2004, Jaffer et al. 2005). Prolonged procedures may be an indicator of perioperative complications, complex surgery, an inexperienced surgical team, less than optimal standardization programs, or patients' pre-existing conditions (Strum et al. 2000, Jibodh et al. 2004, Jaffer et al. 2005).

We found no difference between laminar air flow and conventional operating theater conditions, which is in accordance with the results of several studies questioning the benefits of laminar air flow and which is in line with the idea that deep infection has a multifactorial etiology (Friberg et al. 1998, Brandt et al. 2008, Dale et al. 2009).

Both long- and short-term risk of revision due to infection increased with later calendar time of surgery, which has also been reported recently (Dale et al. 2009). Orthopedic surgeons may be more prone to treat implant infection with surgery or they may be better at registering deep implant infections now than some years ago. In addition, the changes in patient co-morbidity profiles (for example, increased proportion of THA in patients with diabetes and obesity over time) and indications for surgery (e.g. lower threshold for surgery) may in part explain our findings. In the worst case, the true incidence of infection has increased in recent years.

In conclusion, the overall risk of revision due to infection following primary THA surgery in Denmark is less than 1%. Several factors are associated with increased risk, including male sex, co-morbidities, AVN, cement without antibiotic, and hybrid THA, as well as long duration of surgery. AVN and fixation technique did not increase the risk of infection within 1 year of surgery. Further research may help to explain the mechanism underlying this increased risk of infection, which may in turn focus the attention of clinicians on infection prevention strategies.
